# Widespread Sequence Variations in VAMP1 across Vertebrates Suggest a Potential Selective Pressure from Botulinum Neurotoxins

**DOI:** 10.1371/journal.ppat.1004177

**Published:** 2014-07-10

**Authors:** Lisheng Peng, Michael Adler, Ann Demogines, Andrew Borrell, Huisheng Liu, Liang Tao, William H. Tepp, Su-Chun Zhang, Eric A. Johnson, Sara L. Sawyer, Min Dong

**Affiliations:** 1 Department of Microbiology and Immunobiology, Harvard Medical School and Division of Neuroscience, New England Primate Research Center, Southborough, Massachusetts, United States of America; 2 Neurobehavioral Toxicology Branch, U.S. Army Medical Research Institute of Chemical Defense, Aberdeen Proving Ground, Aberdeen, Maryland, United States of America; 3 Department of Molecular Biosciences, University of Texas, Austin, Texas, United States of America; 4 Waisman Center, Department of Neuroscience, Department of Neurology, University of Wisconsin, Madison, Wisconsin, United States of America; 5 Department of Bacteriology, University of Wisconsin, Madison, Wisconsin, United States of America; Boston College, United States of America

## Abstract

Botulinum neurotoxins (BoNT/A-G), the most potent toxins known, act by cleaving three SNARE proteins required for synaptic vesicle exocytosis. Previous studies on BoNTs have generally utilized the major SNARE homologues expressed in brain (VAMP2, syntaxin 1, and SNAP-25). However, BoNTs target peripheral motor neurons and cause death by paralyzing respiratory muscles such as the diaphragm. Here we report that VAMP1, but not VAMP2, is the SNARE homologue predominantly expressed in adult rodent diaphragm motor nerve terminals and in differentiated human motor neurons. In contrast to the highly conserved VAMP2, BoNT-resistant variations in VAMP1 are widespread across vertebrates. In particular, we identified a polymorphism at position 48 of VAMP1 in rats, which renders VAMP1 either resistant (I48) or sensitive (M48) to BoNT/D. Taking advantage of this finding, we showed that rat diaphragms with I48 in VAMP1 are insensitive to BoNT/D compared to rat diaphragms with M48 in VAMP1. This unique intra-species comparison establishes VAMP1 as a physiological toxin target in diaphragm motor nerve terminals, and demonstrates that the resistance of VAMP1 to BoNTs can underlie the insensitivity of a species to members of BoNTs. Consistently, human VAMP1 contains I48, which may explain why humans are insensitive to BoNT/D. Finally, we report that residue 48 of VAMP1 varies frequently between M and I across seventeen closely related primate species, suggesting a potential selective pressure from members of BoNTs for resistance in vertebrates.

## Author Summary

Botulinum neurotoxins (BoNTs) target peripheral motor neurons and act by cleaving SNARE proteins, which are essential for neurotransmitter release from nerve terminals. SNARE proteins occur in multiple homologues and it has been difficult to determine which one is the physiologically relevant toxin target in motor nerve terminals among closely related SNARE homologues such as VAMP1 and VAMP2. Here we report that, in contrast to the highly conserved VAMP2, sequence variations in VAMP1 that confer resistance to BoNTs are widespread across vertebrates. In particular, residue 48 of VAMP1 is polymorphic between BoNT/D-sensitive residue M and BoNT/D-resistant residue I in rats. Taking advantage of this finding, we carried out an intra-species comparison, which showed that diaphragm motor nerve terminals from rats with I48 in VAMP1 were insensitive to BoNT/D as compared to those with M48. Since VAMP2 is conserved in rats, these data demonstrate that VAMP1 is the physiologically relevant toxin target in motor neurons. Interestingly, human VAMP1 encodes the BoNT/D-resistant residue I48, which may explain why humans are insensitive to BoNT/D. Finally, we found that residue 48 of VAMP1 switches frequently between M and I among 17 primate species, suggesting a potential selective pressure from BoNT/D for resistance in primates.

## Introduction

Botulinum neurotoxins (BoNTs) are a family of protein toxins produced by diverse species of *Clostridia*, a genus of anaerobic spore-forming bacteria [1], [2]. These toxins paralyze humans and animals by blocking neurotransmitter release primarily from motor nerve terminals, causing death when respiratory muscles are paralyzed. Adult humans and animals are usually exposed to BoNTs by ingesting preformed toxins in food sources. The disease caused by BoNTs is known as botulism, which has become rare in humans due to improvements in food processing [3]. However, botulism still accounts for large numbers of deaths in both wild and domesticated animals, partly because the clostridia that produce BoNTs are widespread in soils and water sediments [4].

There are seven major serotypes of BoNTs (types A-G) [1], [2]. An eighth serotype (type H) has also been reported recently [5]. These toxins target and enter peripheral nerve terminals via receptor-mediated endocytosis. Once inside neurons, BoNTs translocate across endosomal membranes and act as proteases cleaving one of three proteins essential for synaptic vesicle exocytosis. Specifically, BoNT/B, D, F, and G cleave different sites on the synaptic vesicle protein VAMP (also known as synaptobrevin); BoNT/A, C, and E cleave different sites on the peripheral membrane protein SNAP-25; and BoNT/C also cleaves the plasma membrane protein syntaxin [1], [2]. These three proteins, known as SNARE proteins, form a complex that is the core machinery mediating fusion of synaptic vesicle membranes to plasma membranes [6].

For each of the three SNARE proteins, there are several homologous genes in vertebrate genomes that encode closely related proteins [6]. Previous studies of BoNTs mainly utilized VAMP2, syntaxin 1, and SNAP-25. They are the predominant forms expressed in brain and have been widely used as model SNARE proteins. However, BoNTs cannot cross the blood-brain barrier at concentrations generally encountered during botulism outbreaks. Rather, BoNTs act mainly on peripheral motor neurons and cause death by paralyzing respiratory muscles such as the diaphragm [7]. Therefore, the specific SNARE isoforms expressed in diaphragm motor nerve terminals are the physiological targets that determine the sensitivity of different animal species to BoNTs. SNAP-25 and syntaxin 1 are expressed in motor nerve terminals, and are likely the authentic physiological targets [8], [9], [10]. However, recent data have raised doubts as to whether VAMP2 is a physiologically relevant toxin target in diaphragm motor neurons.

It has been reported that VAMP2 was not detected in rat sciatic motor nerve terminals [11]. Instead, these nerve terminals express VAMP1, another VAMP homologue [11]. It was also recently reported that both VAMP1 and VAMP2 are co-expressed in motor nerve terminals of triangularis sterni muscles in juvenile mice [12]. Furthermore, the lack of VAMP1 expression in a spontaneous VAMP1-null mutant mouse line results in a ∼50% reduction of synaptic transmission in the neuromuscular junctions (NMJs) of juvenile mouse [12]. These VAMP1-null mice exhibit severe motor dysfunction and usually die within 15 days after birth [13]. These phenotypes suggest a possibility that VAMP2 is co-expressed in motor neurons and can partially compensate for the loss of VAMP1 during development, but VAMP1 gradually becomes the dominant form that is essential for survival after 15 days. The expression of VAMP1 relative to VAMP2 in adult rat/mouse diaphragm NMJs has not been examined, and the relative contribution of VAMP1 versus VAMP2 at diaphragm NMJs in adult animals remains unknown.

Interestingly, it has been shown that rat VAMP1 is resistant to BoNT/B in vitro due to a single residue change (Q78V) from the conserved VAMP1 sequence [14], whereas human VAMP1 is a poor substrate for BoNT/D in vitro due to another residue change (M48I) [15]. These in vitro data appear to correlate with the toxicity data in vivo, as it has been a long-standing observation that rats are insensitive to BoNT/B, with a median lethal dose (LD_50_) that is ∼10,000 times the value in mice [16]. Furthermore, it has also been reported that synaptic transmission in isolated human nerve-muscle biopsy tissues was not blocked by BoNT/D [17]. In addition, there is no confirmed human botulism case caused by BoNT/D, despite the prevalence of its association with botulism in domesticated animals [3], [18]. Finally, a recent study of injecting low levels of BoNT/D into the extensor digitorum brevis muscle (EDB) in human volunteers showed no significant paralysis, indicating that humans are indeed insensitive to BoNT/D [19]. Despite these apparent correlations, it is not clear whether the residue changes in VAMP1 underlie the resistance of a species to BoNTs in vivo, and numerous potential confounding factors must be considered when comparing the toxicity of BoNTs in different species.

Here we demonstrate that VAMP1, but not VAMP2, is the predominant form expressed at diaphragm NMJs in adult mice/rats and in human motor neurons differentiated from embryonic stem cells. Furthermore, we found that residue 48 of VAMP1 is polymorphic between M and I in rats. Taking advantage of this finding, we demonstrated that diaphragms from rats homozygous for I48 are insensitive to BoNT/D as compared to diaphragms from rats homozygous for M48. This direct comparison within the same species demonstrates that VAMP1 determines the sensitivity of a species to BoNT/D, and indicates that I48 in human VAMP1 is likely the reason for the insensitivity of humans to BoNT/D. Finally, we report that residue 48 of VAMP1 varies between M and I across seventeen closely related primate species, suggesting a potential selective pressure from BoNT/D.

## Results

### VAMP1, but not VAMP2, is expressed in diaphragm motor nerve terminals in adult rats/mice and in human motor neurons

**Figure 1:**
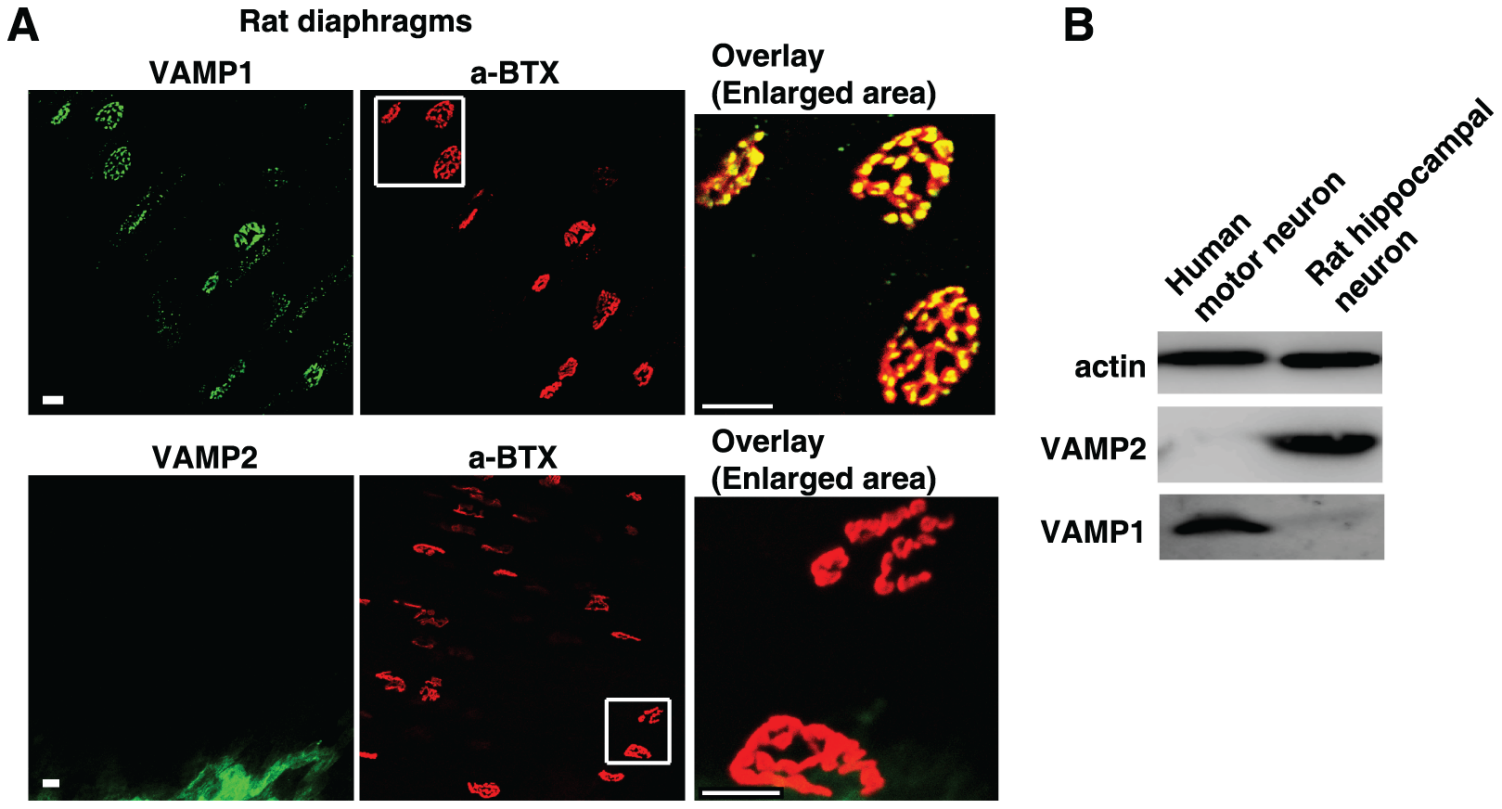
VAMP1, but not VAMP2, is detected at diaphragm motor nerve terminals in adult rats and in human motor neurons. (A) Diaphragms dissected from adult rats were subjected to immunostaining analysis to detect VAMP1 (upper panel) or VAMP2 (lower panel) at NMJs using their specific antibodies. NMJs were labeled with α-BTX. The areas marked by white squares are enlarged to show the overlay between VAMP1 and α-BTX (upper panel), as well as the lack of VAMP2 at NMJs (lower panel). Scale bars represent 20 µm. (B) Cell lysates of human motor neurons differentiated from embryonic stem cells, as well as cultured rat embryonic hippocampal neurons, were subjected to immunoblot analysis to detect VAMP1 and VAMP2 using their specific antibodies. Actin served as a loading control.

Diaphragm motor nerve terminals are the physiological targets that determine species susceptibility to BoNTs. We first examined expression of VAMP1 versus VAMP2 at diaphragm tissues dissected from adult rats and mice. These tissues were fixed, permeabilized, and subjected to immunostaining assays to detect VAMP1 or VAMP2 using their specific antibodies. The diaphragm NMJs were labeled using α-bungarotoxin (α-BTX), which binds to acetylcholine receptors opposing presynaptic motor nerve terminals. We found that VAMP1 is expressed in nearly all NMJs in both rat (Fig. 1A, upper panel) and mouse diaphragms (Fig. S1A, upper panel). VAMP1 largely overlaps with α-BTX at NMJs (Fig. 1A, S1A). In contrast, VAMP2 was not detected under our assay conditions at NMJs in adult rat/mouse diaphragms (Fig. 1A, lower panel; Fig. S1A, lower panel). The VAMP2 antibody utilized here has been widely used for both immunostaining and immunoblot analysis. We also confirmed that this antibody can detect VAMP2 in mouse brain slices following the same fixation and immunostaining protocol used for diaphragm tissues (Fig. S1B).

We next assessed whether VAMP1 is also the dominant form in human motor nerve terminals, utilizing cultured human motor neurons differentiated from embryonic stem cells [20], [21]. Primary rat hippocampal neurons were also assayed as a control. As shown in Fig. 1B, VAMP1 was detected in cell lysates of differentiated human motor neurons, whereas VAMP2 was not detectable under our assay conditions. In contrast, VAMP2 was readily detected in cell lysates of rat hippocampal neurons, which do not express appreciable levels of VAMP1. These results suggest that VAMP1 is likely the dominant form expressed in human motor neurons.

### Rat VAMP1 is resistant to BoNT/B due to a residue change at the toxin cleavage site

**Figure 2:**
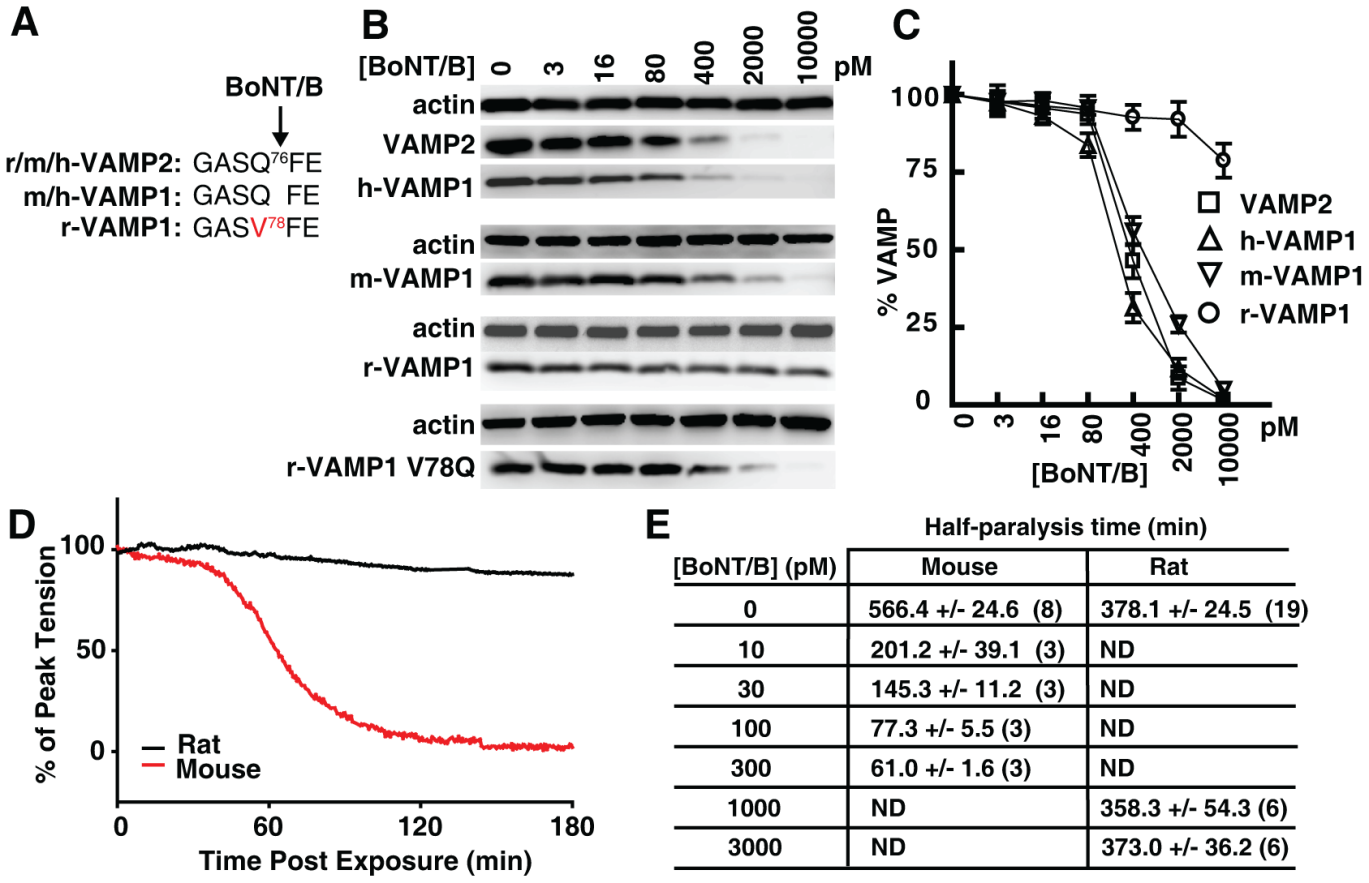
Rat VAMP1 and rat diaphragm motor nerves are insensitive to BoNT/B. (A) The sequence alignment showing the BoNT/B cleavage site within VAMP1 and VAMP2 in indicated species (r: rat; m: mouse; h: human). (B) VAMP1 was expressed in cultured rat hippocampal neurons via lentiviral transduction. Neurons were exposed to the indicated concentrations of BoNT/B for 24 hrs. Cell lysates were collected and subjected to immunoblot analysis to detect VAMP1 and VAMP2. (C) Immunoblot results of VAMP1/2 from panel B were quantified, normalized to actin levels, and plotted to show VAMP1/2 levels after exposure to BoNT/B (N = 3, error bars represent SEM). (D) The effect of BoNT/B on peak twitch tension was determined in isolated mouse and rat phrenic nerve-hemidiaphragm preparations. Representative plots of peak twitch tension with time after exposure to BoNT/B were normalized and compared between mice (red trace, 300 pM) and rats (black trace, 3 nM). (E) Time to attain 50% paralysis (half-paralysis time, mean ± SEM) in rat and mouse phrenic nerve-hemidiaphragm preparations after exposure to BoNT/B. Numbers in parenthesis indicate the number of hemidiaphragms examined. ND: not determined.

It has been a long-held observation that BoNT/B cannot cleave rat VAMP1 in vitro, likely due to a single residue change at the BoNT/B cleavage site (Fig. 2A) [14]. This differential sensitivity between rat VAMP1 and VAMP2 toward BoNT/B provides an opportunity to examine which VAMP homologue plays a dominant role in diaphragm motor nerve terminals.

Because previous reports on the resistance of rat VAMP1 to BoNT/B are based on in vitro studies using either cell lysates or recombinant proteins, we first examined whether rat VAMP1 is indeed resistant to BoNT/B in live neurons. To do so, we utilized primary rat hippocampal neurons as a model. These neurons express VAMP2, but not VAMP1 (Fig. 1B), thus serving as a convenient VAMP1-null neuron model, which allows us to express different versions of VAMP1 exogenously via lentiviral transduction. As shown in Fig. 2B, neurons expressing human (h-), mouse (m-), or rat (r-) VAMP1 were incubated with a gradient of BoNT/B. These neurons were subsequently harvested and subjected to immunoblot analysis to detect cleavage of VAMP2 and VAMP1. Cleavage of VAMP1 and VAMP2 resulted in the loss of their immunoblot signals (Fig. 2B). The remaining VAMP immunoblot signals were normalized to the signal of actin, and subsequently plotted versus the BoNT/B concentrations (Fig. 2C). We found that BoNT/B cleaved VAMP2, h-VAMP1, and m-VAMP1 with similar efficiency, whereas r-VAMP1 was resistant to BoNT/B in neurons (Fig. 2B, C).

We next determined whether the resistance of r-VAMP1 is due to the Q78V residue change at the BoNT/B cleavage site (Fig. 2A). This has been suggested to be the reason, but has not been examined experimentally in neurons. We created a mutated r-VAMP1 in which the V residue at the toxin cleavage site was mutated to Q (Fig. 2A, B). This r-VAMP1 mutant was cleaved by BoNT/B in neurons at a rate similar to m-VAMP1 (Fig. 2B, lower panel), demonstrating that the Q to V change is indeed responsible for the insensitivity of r-VAMP1 to BoNT/B in neurons.

### Differential sensitivities of rat and mouse diaphragm motor nerves to BoNT/B

We next examined whether the difference between rat and mouse VAMP1 may lead to differential sensitivities of their diaphragm motor neurons to BoNT/B. Here we utilized the well-established phrenic nerve-hemidiaphragm preparation, in which the phrenic nerve can be stimulated electrically to trigger muscle contractions. Cleavage of VAMP in phrenic nerve terminals leads to inhibition of synaptic vesicle exocytosis, which blocks muscle contraction.

Using this assay, we found that incubation with 300 pM BoNT/B caused a steep reduction of twitch tension in mouse diaphragm muscles, culminating in nearly complete paralysis within 2 hours (hrs) of toxin addition (Fig. 2D, red line). In contrast, twitch tension in rat diaphragms exposed to a 10-fold higher concentration of BoNT/B (3 nM, Fig. 2D, black line) showed no significant reduction within 2 hrs. The slight reduction observed for rat diaphragms is likely due to a natural run-down of the isolated diaphragm tissues in the bath, since the same level of run-down was observed in control rat diaphragms that were not exposed to BoNT/B.

We further evaluated the sensitivity of rat versus mouse diaphragms to BoNT/B by comparing the time required to produce a 50% reduction of muscle twitch tensions (half-paralysis time) under a series of BoNT/B concentrations. As listed in Fig. 2E, 10 pM of BoNT/B caused a 50% reduction of muscle twitch tension within 201.2±39.1 min on mouse diaphragms. In contrast, rat diaphragms exposed to 1000 pM and 3000 pM concentrations of BoNT/B showed similar rates of reduction of twitch tension as control diaphragms that were not exposed to BoNT/B (∼6 hrs, Fig. 2E), suggesting that rat diaphragm motor nerves are essentially resistant to these levels of BoNT/B. Because VAMP2 is conserved between rats and mice, the dramatically different sensitivities between mouse and rat diaphragms to BoNT/B are consistent with the hypothesis that VAMP1, rather than VAMP2, is the physiologically relevant toxin target in diaphragm motor nerve terminals in adult rats/mice.

### Human VAMP1 is insensitive to BoNT/D due to a residue change at position 48

**Figure 3:**
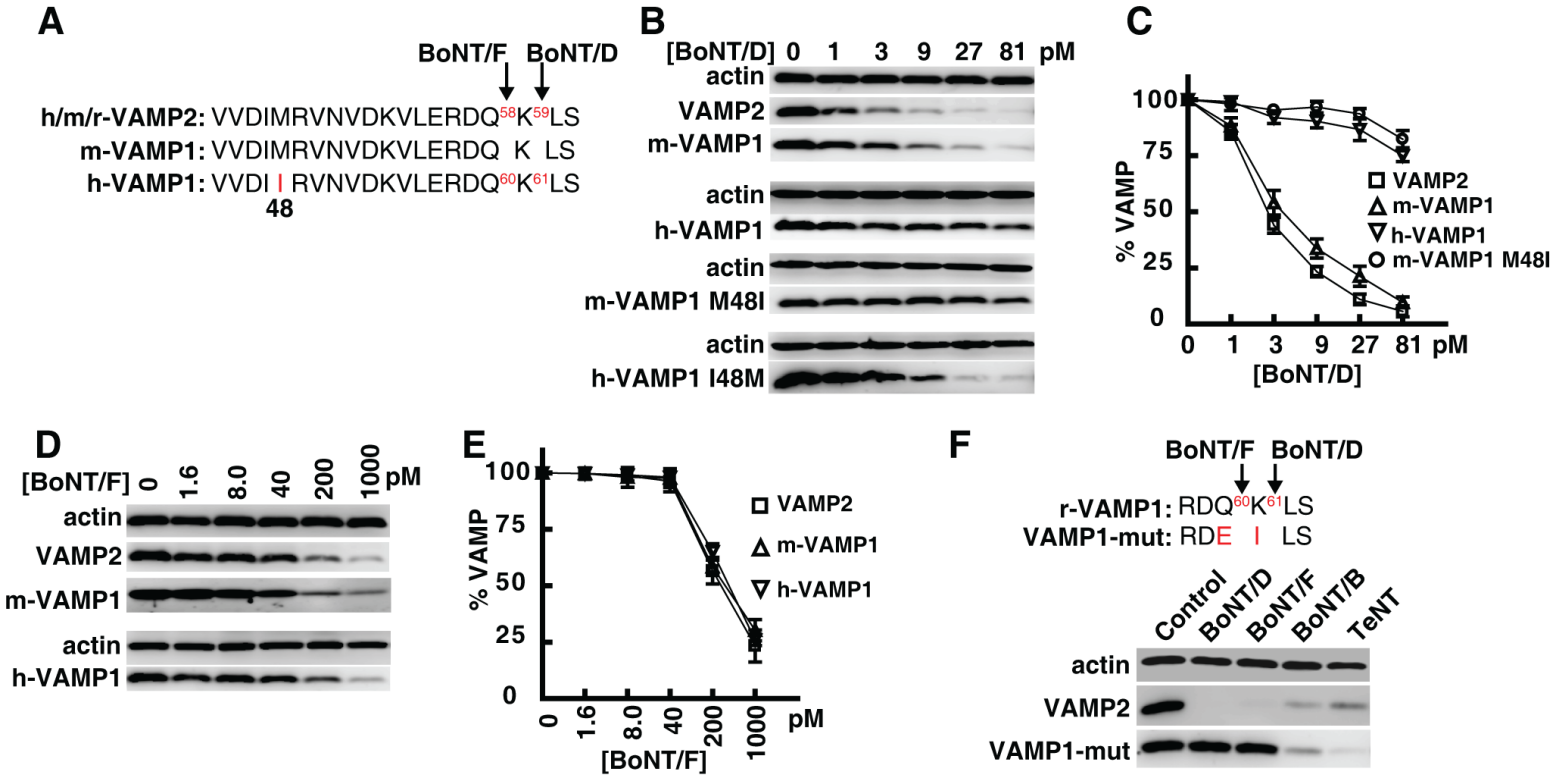
Human VAMP1 is a poor substrate for BoNT/D in neurons. (A) Sequence alignment showing a portion of VAMP1/2, which indicates that human VAMP1 contains residue I at position 48, whereas mouse VAMP1 and VAMP2 both contain residue M at this position. The cleavage sites for BoNT/F and BoNT/D are also designated by arrows. (B) VAMP1 was expressed in cultured rat hippocampal neurons via lentiviral transduction. Neurons were exposed to the indicated concentrations of BoNT/D for 24 hrs. Cell lysates were collected and subjected to immunoblot analysis. Both wild type h-VAMP1 and a mutant m-VAMP1 (M48I) were poorly cleaved by BoNT/D, whereas wild type m-VAMP1 and a mutant h-VAMP1 (I48M) were efficiently cleaved by BoNT/D. (C) Immunoblot results from panel B were quantified and plotted (N = 3, error bars represent SEM). (D) Experiments were carried out as described in panel B, except that neurons were exposed to BoNT/F. Both m- and h-VAMP1 were cleaved by BoNT/F at similar rates. (E) Quantification of immunoblot results from panel D (N = 3, error bars represent SEM). (F) A mutant m-VAMP1 (VAMP1-mut) containing Q60E/K61I was expressed in cultured rat hippocampal neurons via lentiviral transduction. Cells were exposed to the indicated toxins for 24 hrs, and cell lysates were subjected to immunoblot analysis to detect cleavage of VAMP1. Cleavage of endogenous VAMP2 serves as an internal control to demonstrate the activity of toxins. VAMP1 Q60E/K61I was resistant to both BoNT/F (1 nM) and BoNT/D (1 nM), but was readily cleaved by BoNT/B (2 nM) and TeNT (0.15 nM).

It has been reported that human VAMP1 is insensitive to BoNT/D in vitro, due to another single residue change at position 48 (M48I, Fig. 3A) [15]. Although position 48 is outside the cleavage site for BoNT/D, it is within the motif where BoNT/D binds and thus may affect the ability of BoNT/D to recognize and bind VAMP1 efficiently [22].

We first evaluated whether human VAMP1 is indeed resistant to BoNT/D in live neurons. As shown in Fig. 3B, primary rat hippocampal neurons expressing human or mouse VAMP1 were incubated with BoNT/D. Cleavage of VAMP1 and VAMP2 was determined via immunoblot analysis of the neuron lysates. We found that human VAMP1 was much less sensitive to BoNT/D than mouse VAMP1 in neurons (Fig. 3B, C). Furthermore, mutation of M48I in m-VAMP1 caused it to become insensitive to BoNT/D, whereas mutation of I48M in h-VAMP1 allowed it to be cleaved at a rate similar to wild type m-VAMP1 (Fig. 3B, C). In contrast to BoNT/D, BoNT/F cleaved m-VAMP1, h-VAMP1, and endogenous VAMP2 at similar rates (Fig. 3D, E), indicating that I48 residue specifically reduces the cleavage efficacy of BoNT/D. Together, these data confirmed that human VAMP1 is insensitive to BoNT/D in neurons due to a residue change at position 48.

### Widespread residue changes in VAMP1 at BoNT cleavage sites across vertebrates

We next evaluated whether toxin-resistant residue changes occur in other species, by examining VAMP1 sequences available in public databases. We found that the same residue change as in rats (Q78V) also occurs in chickens and finches (Fig. S2). The M48I residue change is even more widespread, including in several primates, as well as in the rat, torpedo, wild boar, Tasmanian devil, and elephant (Fig. S2).

In addition to these two sites, we also found residue changes in VAMP1 at the cleavage sites for BoNT/F and BoNT/D (Q60E, K61I, Fig. 3F, S2), in species such as the cat, dog, horse, and panda. Introducing these two residue changes into m-VAMP1 renders it resistant to both BoNT/D and /F in neurons (Fig. 3F). This mutant VAMP1 can still be cleaved by BoNT/B and tetanus neurotoxin (TeNT), demonstrating the specificity of its resistance to BoNT/D and /F (Fig. 3F). As shown in Fig. S2, additional residue changes in VAMP1 also exist at the cleavage site for BoNT/F5 (E57K). In contrast, VAMP2 from these species showed no residue changes at the BoNT cleavage sites (Fig. S3).

### Position 48 of VAMP1 is polymorphic between M and I residues in rats

**Figure 4:**
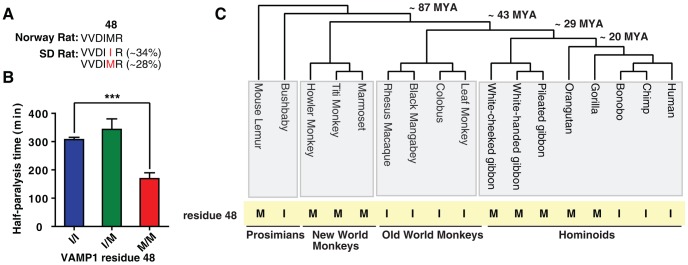
M/I48 residue changes exist across a broad range of primate species and determine the species sensitivity to BoNT/D. (A) Norway rats contain M at position 48 of VAMP1, whereas VAMP1 amplified from a group of 35 SD rats revealed that 34% are homozygous with residue I, 28% are homozygous with residue M, and the rest are I/M heterozygous at position 48 of VAMP1. (B) Muscle twitch tension was recorded from rat phrenic nerve-hemidiaphragm preparations as described in Fig. 2D, except that muscles were exposed to BoNT/D (300 pM). Muscles were dissected from adult SD rats without foreknowledge of their genotypes. Rat tissues were genotyped afterwards to determine the specific residue at position 48 of VAMP1. The half-paralysis time was grouped based on the genotype of VAMP1 (I/I: 307±7 min; I/M: 343±37 min; M/M: 169±21 min; N = 4; the error bars represent SEM). The difference between I and M groups was significant (P<0.0008, two tail t-test), whereas the difference between I and I/M was not significant (P = 0.38). (C) The residues at position 48 of VAMP1 in seventeen different primate species are indicated. These primate species represent ∼87 million years (MYA) of primate evolution, covering four major primate groups as indicated. The residue at position 48 is highly variable between different primate species and toggles frequently between M and I residues (highlighted in yellow).

The finding that human VAMP1 is insensitive to BoNT/D is consistent with previous observations that humans are less sensitive to BoNT/D [19]. However, whether the former is the reason for the latter has not been established. As rat VAMP1 has been reported to also contain residue I at position 48 and is resistant to BoNT/D in vitro [23], [24], rats may serve as a model to investigate whether VAMP1 with I48 can confer resistance to BoNT/D in diaphragm motor nerve terminals. Surprisingly, our search of public sequence databases revealed an additional rat VAMP1 sequence with residue M at position 48. Further analysis revealed that residue M was reported from the genome sequence of Norway rats, whereas residue I was reported for VAMP1 cloned from Sprague Dawley (SD) rats (Fig. 4A), and this is the only difference in VAMP1 between these two types of rats. Therefore, residue 48 can be either M or I among different rat species. The SD rat is a widely utilized laboratory strain. As this is an outbred strain, we next examined whether residue 48 could be polymorphic even within SD rats. To answer this question, we sequenced VAMP1 from a total of 35 adult SD rats purchased from a commercial vendor (Charles River). The results revealed that position 48 is the only polymorphic residue, with 34% of SD rats homozygous with I residue and 28% homozygous with M, while the rest were M/I heterozygous (Fig. 4A).

### An intra-species comparison demonstrates that VAMP1 determines the sensitivity of rat diaphragm motor nerves to BoNT/D

The finding that VAMP1 in the SD rat can have either M or I at position 48 provides an ideal opportunity to address the question of whether the M/I residue change in VAMP1 underlies species sensitivity to BoNT/D. By comparing SD rats with different genotypes in VAMP1, we can significantly reduce, if not eliminate, any potential confounding factors that are always a concern when comparing different species.

We thus examined the sensitivity of diaphragms from SD rats to BoNT/D, using the phrenic nerve-hemidiaphragm tissue preparation described in Fig. 2D. These rats were ordered without foreknowledge of their genotypes, subjected to our assay, and then genotyped afterwards. Thus, the experimenter who measured the sensitivity of each rat diaphragm to BoNT/D was blind to the specific genotype of that rat. As the assay was done blindly, we only compared the sensitivity of rat diaphragms to a fixed BoNT/D concentration, 300 pM. As shown in Fig. 4B, rats that were homozygous with residue M exhibited a half-paralysis time of 169±21 min; whereas rats that were homozygous with residue I had a half-paralysis time of 307±8 min. The heterozygous rats with M/I exhibited the same level of insensitivity as the homozygous rats with residue I (with a half-paralysis time of 343±37 min). These data demonstrate that M/I residues at position 48 of VAMP1 largely determine the sensitivity of the diaphragm motor nerve terminals to BoNT/D.

### Frequent M/I switches at position 48 of VAMP1 among different primate species

The diversity of primate species provides an ideal model for us to further survey toxin-resistant residue changes in VAMP1 among closely related species. For this purpose, we selected seventeen major primate species from four primate groups, which represent ∼87 million years (MYA) in primate evolution (Fig. 4C). For several of the primates, VAMP1 sequences were available from previous genomic studies. For the ones that were not available, we sequenced the first four exons of their VAMP1 gene, encoding residues 1–113.

All but 4 of these 113 amino acid positions in VAMP1 were perfectly conserved across these seventeen primates. Two of the variable amino acid positions, positions 7 and 106, differed in only a single species (Fig. S4, P7L in Titi Monkey, and A106T in Colobus). In contrast, the other two positions, residues 28 and 48, changed multiple times among primates. Residue 28 varies among three amino acids: M/L/T (Fig. S4), while residue 48 toggles between M and I (Fig. 4C). As a control, we also analyzed VAMP2 sequences (residues 1–116) in nine major primate species (Fig. S5). In contrast to VAMP1, VAMP2 is 100% conserved in all nine primate species.

The functional significance of changes at residue 28 of VAMP1 is unknown, but it is unlikely to be related to BoNT sensitivity since residue 28 is outside of both toxin recognition and cleavage sites [22]. Residues at position 48, however, would determine the susceptibility of a particular primate species to BoNT/D as we demonstrated using the SD rat model (Fig. 4B). The distribution of M/I at position 48 shows a tentative link to the geographic distribution of primates. As shown in Fig. S6, all three primates tested from the Americas encode the toxin-sensitive M residue, whereas almost all African primates encode the toxin resistant I residue. Primates sampled from Asia encode a mixture of the two. Humans encode the toxin-resistant I, as do the majority of African primates, which is in line with human emergence from the African continent.

## Discussion

VAMP1 and VAMP2 are two highly homologous synaptic vesicle SNARE proteins. They exhibit largely distinct expression patterns in the central and peripheral nervous systems, but also overlap in many regions [11], [25], [26], [27], [28]. Both are substrates for BoNT/B/D/F/G [1], but VAMP2 has been the prevalent homologue utilized in the majority of studies on BoNT action. This is partly because VAMP2 has been widely utilized as a model VAMP protein. However, the specific VAMP isoform that is the physiologically relevant toxin target in diaphragm motor nerve terminals has not been established.

Here we examined the expression of VAMP1 versus VAMP2 using their specific antibodies, which showed that VAMP1 is the predominant isoform expressed in diaphragm NMJs in adult mice/rats. We further examined the role of VAMP1/VAMP2 in diaphragms at functional levels. First, we confirmed that rat diaphragms are insensitive to BoNT/B. This is consistent with a dominant role for VAMP1 at diaphragms, since rat VAMP1 is resistant to BoNT/B, whereas rat VAMP2 is readily cleaved by BoNT/B. Second, we found that rats are polymorphic at residue 48 of VAMP1 between M that is sensitive to cleavage by BoNT/D, and I that is a poor substrate for BoNT/D. Taking advantage of this finding, we demonstrated that the sensitivity of rat diaphragm motor nerves to BoNT/D correlates with the specific residue 48 in VAMP1: diaphragms from rats with I48 were much less sensitive to BoNT/D than diaphragms from rats with M48. This unique intra-species comparison provides strong evidence that VAMP1 is the dominant form in adult rat diaphragm motor nerve terminals. This is likely also the case in humans, as we showed that cultured human motor neurons differentiated from embryonic stem cells mainly express VAMP1, but not VAMP2.

Our mutagenesis studies further validated in neurons that the resistance of rat VAMP1 to BoNT/B is due to the presence of residue V at position 78, whereas the resistance of human VAMP1 to BoNT/D is due to residue I at position 48. Interestingly, these residue changes occur in multiple vertebrate species. In particular, residue 48 is a hot spot that switches frequently between BoNT/D-sensitive residue M and BoNT/D-insensitive residue I across closely related primate species. Residue 48 is one of the key residues within the highly conserved SNARE domain of VAMP1/2, directly participating in the formation of the SNARE helix bundle [29], [30]. It is also a key position critical for endocytosis of VAMP2 and its localization onto vesicles [31], [32]. Because this M/I switch occurs within closely related primate species, it is not likely due to an evolutionary selection for any cellular functions. Although we cannot exclude the possibility that the M/I switch is a random and neutral event, the finding that this single change determines the sensitivity of the species to BoNT/D suggests that BoNT/D might have exerted a selective pressure on primates.

We were surprised to find that the M/I residue change also occurs within the commonly utilized SD rats. It is a remarkable example that a single residue polymorphism in animal models exhibits a dramatic impact on their susceptibility to toxins/pathogens. Consequently, it is essential to determine the specific VAMP1 genotype of each SD rat when using this animal model for studying BoNT/D action. Whether similar polymorphisms exist in other species and humans remains to be resolved.

As low levels of BoNT/D selectively cleave human VAMP2, but not VAMP1, it might be a unique toxin that can attenuate synaptic transmission in VAMP2-expressing neurons in humans, without the danger of causing muscle paralysis. Therefore, it will be interesting to establish the distribution of VAMP1 versus VAMP2 in neurons other than motor neurons, such as sensory neurons involved in pain pathways. It has been reported that both VAMP1 and VAMP2 are expressed in trigeminal ganglion neurons involved in migraine [33]. VAMP2 is also reported to be expressed in the dorsal horn nerve terminals and in small- and medium-sized dorsal root ganglion neurons [11]. The population of SD rats with I48 may provide a convenient model to explore the therapeutic potential of using BoNT/D to target VAMP2-expressing cells. Finally, our findings of frequent M/I changes among primate species also provide crucial guidance for selecting appropriate primate models for understanding the toxicity and therapeutic effects of BoNT/D in humans.

## Materials and Methods

### Ethics statement

This study was carried out in strict accordance with the recommendations in the Guide for the Care and Use of Laboratory Animals of the National Institutes of Health. The animal protocol was approved by the Standing Committee on Animals of Harvard Medical School (Protocol Number: 04619). For experiments carried out at the USAMRICD, the experimental protocol was approved by the Animal Care and Use Committee at the USAMRICD (Protocol Number: 109U949). All procedures were conducted in accordance with the principles stated in the Guide for the Care and Use of Laboratory Animals and the Animal Welfare Act of 1966 (P.L. 89–544), as amended. All efforts were made to minimize suffering of animals.

This study utilized a NIH-approved human embryonic stem cell line (H9) for differentiation into human motor neurons, and it has been approved by the Harvard Embryonic Stem Cell Research Oversight (ESCRO) Committee (Protocol Number: E00030). The identity of the donor of the embryo is anonymous and cannot be readily ascertained by the investigator. It is therefore not considered human subjects research and does not require IRB review.

### Antibodies, toxins, cDNAs and constructs

Mouse monoclonal antibodies for VAMP2 (69.1) were generously provided by E. Chapman (Madison, WI). The following antibodies and reagents were purchased from the indicated vendors: mouse monoclonal antibodies for actin (AC-15, Sigma-Aldrich), rabbit polyclonal antibodies against synapsin (Millipore) and VAMP1 (Synaptic Systems), Alexa-594 conjugated α-Bungarotoxin (α-BTX, Invitrogen). BoNT/B (Okra), BoNT/D (D5995), and BoNT/F (Langeland) were either purchased from Metabiologics (Madison, WI) or provided by E. Johnson (Madison, WI). The cDNA encoding mouse VAMP1 was generously provided by C. Hu (Louisville, KY). The cDNAs encoding human and rat VAMP1 were purchased from Open Biosystems (Waltham, MA). The full-length VAMP1 was sub-cloned into Lox-Syn-Syn lentiviral vectors, which contain two separate neuronal specific synapsin promoters, one drives expression of VAMP1, the other drives expression of GFP as a marker. Mutagenesis studies were carried out using the QuickChange Site-directed Mutagenesis kit (Agilent Technologies).

### Neuron culture and lentivirus preparations

Primary rat hippocampal neurons were prepared from E18-19 embryos using a papain dissociation kit (Worthington Biochemical, NJ), as we described previously [34]. Lentiviruses were prepared using packaging vectors VSV-G and Δ8.9 [34]. Viruses were added to DIV 5 (days in vitro) neurons. Experiments were carried out at DIV 14–16. This study was carried out in strict accordance with the animal protocol approved by the Standing Committee on Animals of Harvard Medical School (Protocol Number: 04619). All procedures were conducted in accordance with the principles stated in the Guide for the Care and Use of Laboratory Animals and the Animal Welfare Act of 1966 (P.L. 89–544), as amended.

### Immunoblot analysis

Neurons were washed and lysed using RIPA buffer (50 mM Tris, 1% NP40, 150 mM NaCl, 0.5% sodium deoxycholate, 0.1% SDS) with a protease inhibitor cocktail (Sigma-Aldrich). Cell lysates were centrifuged for 10 min at 4°C. Supernatants were subjected to western blot analysis using the enhanced chemiluminescence (ECL) method (Pierce). The ECL signals were collected using a Fuji LAS 3000 digital darkroom and quantified using ImageJ software. The following antibody dilutions were used in these assays: anti-VAMP1 (1∶500); anti-VAMP2 (1∶1000); anti-actin (1∶1000).

### Nicking of BoNT/B

Since BoNT/B contains a mixed population of single chain (inactive form) and di-chain (active form) toxins, it was activated (nicked) by limited proteolysis to obtain a uniform population of di-chain toxins. Briefly, BoNT/B (6.6 µM) was diluted to 800 nM with 30 mM HEPES (pH 6.8) and incubated at 37°C for 30 min with bovine pancreatic trypsin type XI (13 µM). The reaction was stopped by addition of an excess of soybean trypsin inhibitor (23 µM, type I-S) for 15 min. Bovine serum albumen (0.1%) was then added for stability, and the nicked toxin was dispensed in single use vials and stored at −20°C until use.

### Mouse/rat phrenic nerve-hemidiaphragm preparations

Experiments were performed on isolated hemidiaphragm muscles dissected from adult male Crl:CD1(ICR) mice (19–24 g) and adult SD rats (250–400 g) (Charles River, Wilmington, MA). Animals were euthanized by decapitation following exposure to excess isoflurane. Hemidiaphragms with attached phrenic nerves were mounted in 20-ml (mouse diaphragm) or 60-ml (rat diaphragm) tissue baths containing Tyrode's solution of the following composition (mM): NaCl, 137; KCl, 5; MgSO_4_, 1; NaHCO_3_, 24; NaH_2_PO_4_, 1; CaCl_2_, 1.8, and glucose, 11. The solution was bubbled with a gas mixture of 95% O_2_/5% CO_2_ yielding a pH of 7.3–7.4. Resting tensions were maintained at 0.7 g (mouse diaphragm) or 2.0 g (rat diaphragm) to generate optimal nerve-evoked contractions. This study was carried out in strict accordance with the animal protocol approved by the Animal Care and Use Committee at the USAMRICD (Protocol Number: 109U949), and all procedures were conducted in accordance with the principles stated in the Guide for the Care and Use of Laboratory Animals and the Animal Welfare Act of 1966 (P.L. 89–544), as amended.

### Human motor neuron differentiation

Human motor neurons were differentiated as previously described [20], [21]. Briefly, embryonic stem cells (hESCs, lines H9, passages 19 to 35) were used to generate neuroectodermal cells. Derived neuroectodermal cells were treated with retinoic acid (0.1 µM) for caudalization in neural medium (NM: DMEM/F12, nonessential amino acids, 2 µg/ml heparin, and the neural cell supplement N2). The neuroepithelial clusters were isolated and suspended in the NM in the presence of both retinoic acid and purmorphamine (1 µM). The formed progenitor spheres were subsequently cultured on glass coverslips coated with polyornithine and laminin (2–4 clusters/coverslip in a 24-well plate) in the presence of 0.5 ml NM, supplemented with BDNF (10 ng/ml; Peprotech), GDNF(10 ng/ml; R&D Systems), IGF1 (10 ng/ml; Peprotech), cAMP (1 µM), ascorbic acid (200 ng/ml, Innovative Cell Technology), and 50 nM retinoic acid. It took 28 days to differentiate embryonic stem cells into motor neurons. These neurons were further cultured for 7 weeks to reach the stage that is considered to be mature for motor neurons in culture. Thus, the age of human motor neurons used in our experiments is 11 weeks old from the original stem cells.

### Sequencing VAMP1 from SD rats

Genomic DNA was extracted from rat tails. A 170 base pair fragment that covers position 48 in the VAMP1 sequence was amplified by PCR using the following primers: 5′-CCTGGCTTTCGATCTCT-3′, and 5′-TCAGCATCTTTATTCCTGC-3′. The PCR fragments were then purified and sequenced.

### Immunostaining of diaphragm NMJs

Diaphragms were dissected from adult rats/mice (2-months-old) and fixed overnight with 2% paraformaldehyde (PFA). Tissues were incubated with α-BTX (1 µg/ml for 60 min) and then permeabilized with 0.5% Triton X-100 for 30 min. Tissues were further incubated with the blocking buffer (PBS with 3% BSA, 0.5 M NaCl and 0.3% Triton X-100) for 30 min, and then treated with 0.2% citric acid for 30 min, washed with PBS, and incubated with primary antibody diluted in blocking buffer (anti-VAMP1: 1∶300; anti-VAMP2: 1∶200) overnight at 4°C, rinsed and further incubated with Alexa-488 conjugated secondary antibody for 2 hrs at room temperature. Samples were washed and mounted. Images were acquired using a confocal microscope (Leica SP5, 10× objective), and presented as maximum projections from Z-stack acquisitions.

### Immunostaining of brain slices

Wild type C57BL/6 mice were anesthetized with 2% avertin (20 µl/g) and transcardially perfused with 4% PFA. The whole brain was isolated and fixed in 4% PFA for 24 hrs, rinsed with PBS, and immersed in 30% sucrose in PBS. Coronal brain sections were cut at 30 µm segments with a freezing sliding microtome. Brain slices were subjected to immunostaining analysis with antibodies against VAMP2 (1∶200) and synapsin (1∶400).

### Muscle tension recording

Muscles were stimulated by application of supramaximal volleys to the phrenic nerve via bipolar stainless steel electrodes (6.0–9.0 V, 0.2 millisecond duration) at 0.033 Hz. Muscle tensions were measured using Grass FT03 force displacement transducers (Astro-Med, Inc., West Warwick, RI), digitized and analyzed offline using pClamp software V10 (Molecular Devices, Sunnyvale, CA). Tissues were maintained at 36°C and exposed to BoNTs by adding toxins directly to the incubation solution.

### Sequencing primate VAMP1/VAMP2

Chimpanzee, gorilla, orangutan, white-cheeked gibbon, rhesus macaque, marmoset, bushbaby and mouse lemur gene sequences were obtained for both VAMP1 and VAMP2 from the UCSC genome database (http://genome.ucsc.edu/). Vamp1 was sequenced from eight additional primate species from primate cell lines as previously described [35]. The VAMP1 sequence was amplified by PCR from cDNA using the primer pairs: 5′-CAGCCTCCGGAGAGGAAC-3′ with 5′-GCTTCATTTGACTGCAAAGTCCC-3′ or 5′-TGTCTCGCCGCAGCCTC-3′ with 5′-GTAAATATGCCACATGCCTTTGGTGG-3′. The PCR fragments were then sequenced using the PCR primers listed above or the following primers: 5′-TTACAATAACTACCACGATGATGGC-3′ or 5′-CCAGAGGAGAGTGGAGACC-3′. Primate VAMP1 gene sequences have been deposited in GenBank (accession numbers KJ720564–KJ720571).

## Supporting Information

Figure S1 **VAMP2 expression can be detected in mouse brain slices, but not in diaphragm motor nerve terminals in adult mice.** (A) Diaphragms dissected from adult mice were subjected to immunostaining analysis to detect VAMP1 (upper panel) and VAMP2 (lower panel) at motor nerve terminals using their specific antibodies. NMJs were labeled with α-BTX. Scale bars represent 20 µm. (B) Mice were fixed by perfusion with 4% PFA. Coronal brain slices were subjected to immunostaining analysis with the same VAMP2 antibody as used in panel A. Synapsin served as a marker for presynaptic terminals. VAMP2 was detected in brain slices and was largely co-localized with synapsin. The scale bar represents 10 µm.
(PDF)

Figure S2 **Sequence alignment of VAMP1 in selected vertebrate species.** Selected vertebrates were organized based on their phylogenetic relationship. Their VAMP1 residues at the cleavage site for each BoNT (position 56/57 for BoNT/F5, position 60/61 for BoNT/F and D, position 78 for BoNT/B, and position 83/84 for BoNT/G) were aligned, together with the residue at position 48. Residues that differ from the conserved sequence are highlighted in red.
(PDF)

Figure S3 **Sequence alignment of VAMP2 in selected vertebrate species.** Sequence alignment of VAMP2 from selected vertebrate species shows that VAMP2 is highly conserved, with no residue changes at any site examined.
(PDF)

Figure S4 **Sequence alignment of VAMP1 in selected primate species.** Protein sequences of exons 1–4 of the VAMP1 gene are aligned for the seven listed primate species. Out of the 113 amino acid positions shown, only four positions have accumulated non-synonymous mutations. Positions 7 and 106 have a single non-synonymous mutation in only one primate species (highlighted in green), whereas sites 28 and 48 are highly variable, having mutated multiple times over primate evolution (highlighted in yellow).
(PDF)

Figure S5 **Sequence alignment of VAMP2 in selected primate species.** There are no non-synonymous mutations in VAMP2 across nine major primate species examined here, indicating that VAMP2 is highly conserved.
(PDF)

Figure S6 **Geographic distribution of major primate species.** The geographic distribution of all seventeen primate species examined in Fig. 4C was color-coded and plotted on the map. The primates with M48 in VAMP1 are marked in blue, whereas the primates with I48 in VAMP1 are marked in red.
(PDF)
