# Supplementary material for: Widespread Sequence Variations in VAMP1 across Vertebrates Suggest a Potential Selective Pressure from Botulinum Neurotoxins
Source: PLoS Pathog. 2014 Jul 10;10(7):e1004177. doi: 10.1371/journal.ppat.1004177 (PMC4092145; doi:10.1371/journal.ppat.1004177)
Supplement: Figure S1 — VAMP2 expression can be detected in mouse brain slices, but not in diaphragm motor nerve terminals in adult mice. (A) Diaphragms dissected from adult mice were subjected to immunostaining analysis to detect VAMP1 (upper panel) and VAMP2 (lower panel) at motor nerve terminals using their specific antibodies. NMJs were labeled with α-BTX. Scale bars represent 20 µm. (B) Mice were fixed by perfusion with 4% PFA. Coronal brain slices were subjected to immunostaining analysis with the same VAMP2 antibody as used in panel A. Synapsin served as a marker for presynaptic terminals. VAMP2 was detected in brain slices and was largely co-localized with synapsin. The scale bar represents 10 µm. (PDF) [file ppat.1004177.s001.pdf]

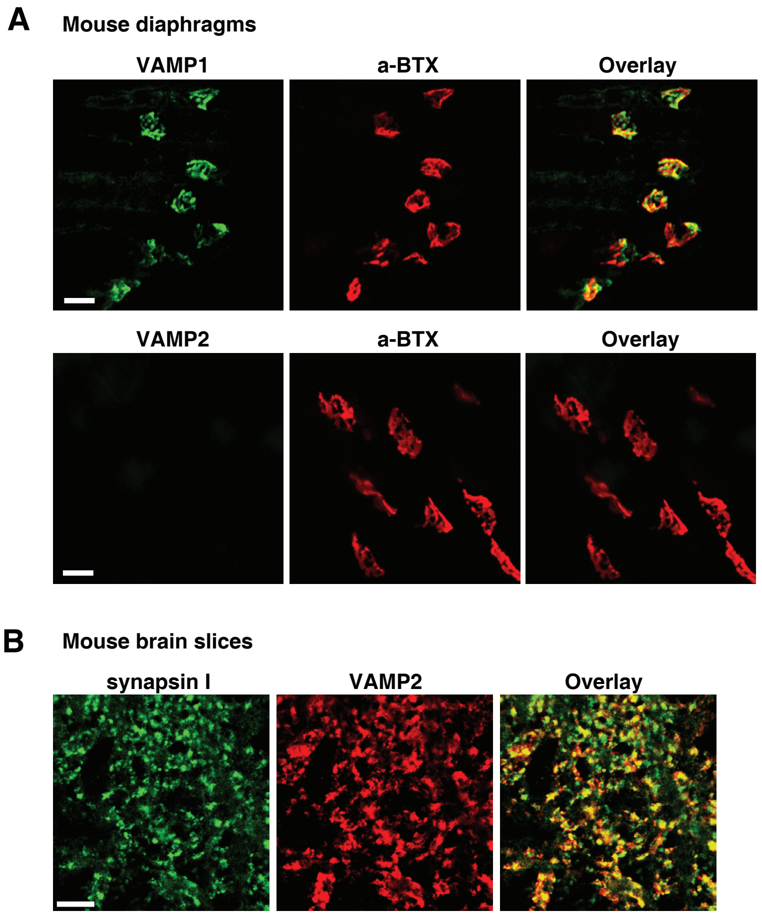


**Supplementary Figure 1. VAMP2 expression can be detected in mouse brain slices, but not in diaphragm motor nerve terminals in adult mice.**

(A) Diaphragms dissected from adult mice were subjected to immunostaining analysis to detect VAMP1 (upper panel) and VAMP2 (lower panel) at motor nerve terminals using their specific antibodies. NMJs were labeled with α-BTX. Scale bars represent 20 µm.

(B) Mice were fixed by perfusion with 4% PFA. Coronal brain slices were subjected to immunostaining analysis with the same VAMP2 antibody as used in panel A. Synapsin served as a marker for presynaptic terminals. VAMP2 was detected in brain slices and was largely co-localized with synapsin. The scale bar represents 10 µm.
