# Supplementary material for: Widespread Sequence Variations in VAMP1 across Vertebrates Suggest a Potential Selective Pressure from Botulinum Neurotoxins
Source: PLoS Pathog. 2014 Jul 10;10(7):e1004177. doi: 10.1371/journal.ppat.1004177 (PMC4092145; doi:10.1371/journal.ppat.1004177)
Supplement: Figure S2 — Sequence alignment of VAMP1 in selected vertebrate species. Selected vertebrates were organized based on their phylogenetic relationship. Their VAMP1 residues at the cleavage site for each BoNT (position 56/57 for BoNT/F5, position 60/61 for BoNT/F and D, position 78 for BoNT/B, and position 83/84 for BoNT/G) were aligned, together with the residue at position 48. Residues that differ from the conserved sequence are highlighted in red. (PDF) [file ppat.1004177.s002.pdf]

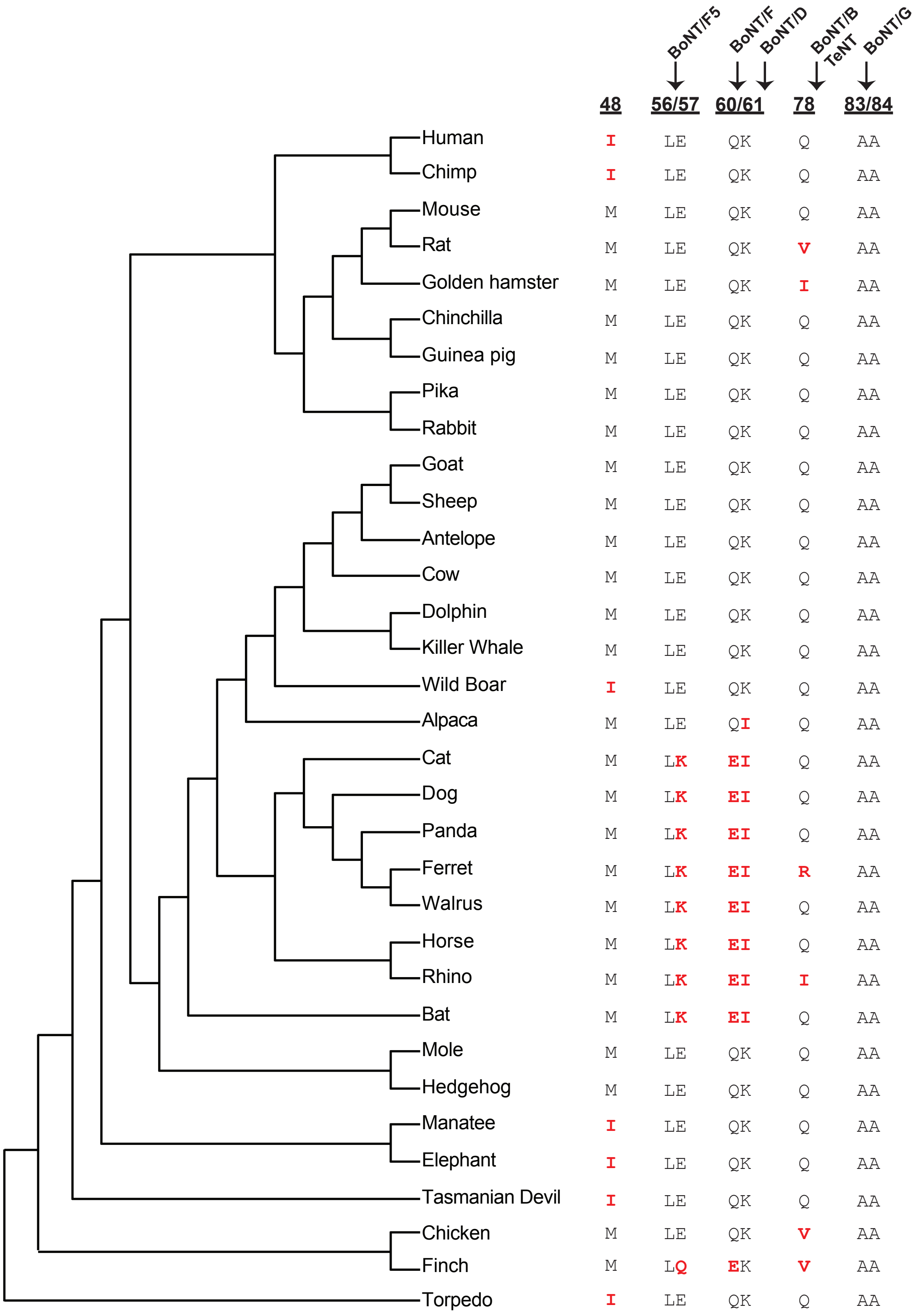

Supplementary Figure 2. Sequence alignment of VAMP1 in selected vertebrate species.

Selected vertebrates were organized based on their phylogenetic relationship. Their VAMP1 residues at the cleavage site for each BoNT (position 56/57 for BoNT/F5, position 60/61 for BoNT/F and D, position 78 for BoNT/B, and position 83/84 for BoNT/G) were aligned, together with the residue at position 48. Residues that differ from the conserved sequence are highlighted in red.
