# Supplementary material for: Widespread Sequence Variations in VAMP1 across Vertebrates Suggest a Potential Selective Pressure from Botulinum Neurotoxins
Source: PLoS Pathog. 2014 Jul 10;10(7):e1004177. doi: 10.1371/journal.ppat.1004177 (PMC4092145; doi:10.1371/journal.ppat.1004177)
Supplement: Figure S3 — Sequence alignment of VAMP2 in selected vertebrate species. Sequence alignment of VAMP2 from selected vertebrate species shows that VAMP2 is highly conserved, with no residue changes at any site examined. (PDF) [file ppat.1004177.s003.pdf]

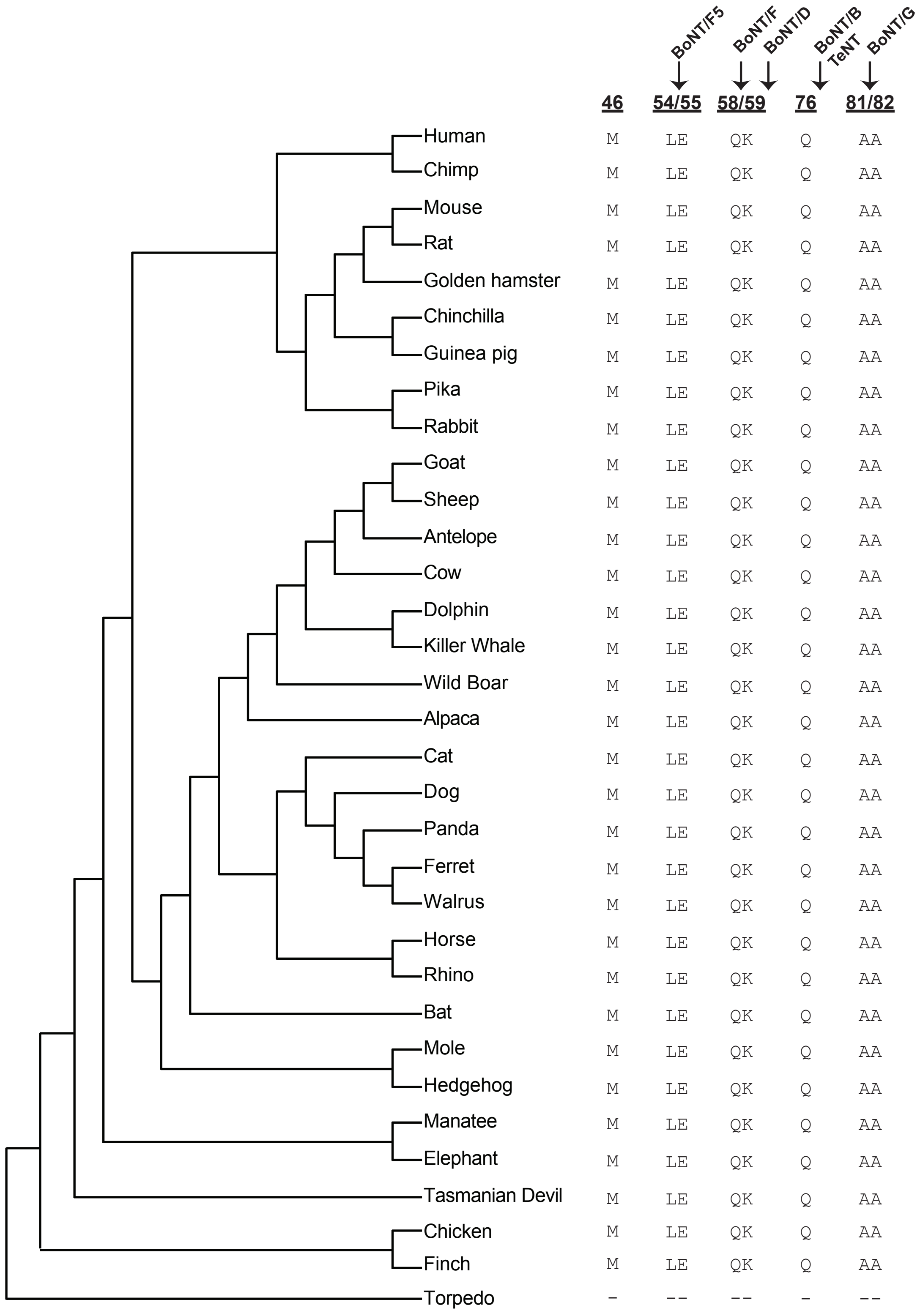

Supplementary Figure 3. Sequence alignment of VAMP2 in selected vertebrate species.

Sequence alignment of VAMP2 from selected vertebrate species shows that VAMP2 is highly conserved, with no residue changes at any site examined.
