# Supplementary material for: Widespread Sequence Variations in VAMP1 across Vertebrates Suggest a Potential Selective Pressure from Botulinum Neurotoxins
Source: PLoS Pathog. 2014 Jul 10;10(7):e1004177. doi: 10.1371/journal.ppat.1004177 (PMC4092145; doi:10.1371/journal.ppat.1004177)
Supplement: Figure S4 — Sequence alignment of VAMP1 in selected primate species. Protein sequences of exons 1–4 of the VAMP1 gene are aligned for the seven listed primate species. Out of the 113 amino acid positions shown, only four positions have accumulated non-synonymous mutations. Positions 7 and 106 have a single non-synonymous mutation in only one primate species (highlighted in green), whereas sites 28 and 48 are highly variable, having mutated multiple times over primate evolution (highlighted in yellow). (PDF) [file ppat.1004177.s004.pdf]

```
                            7                    28                  48
Human                  MSAPAQPPAEGTEGTAPGGGPPGPPPNMTSNRRLQQTQAQVEEVVDIIRVNVDKVLERDQ
Chimp                  MSAPAQPPAEGTEGTAPGGGPPGPPPNMTSNRRLQQTQAQVEEVVDIIRVNVDKVLERDQ
Bonobo                 MSAPAQPPAEGTEGTAPGGGPPGPPPNMTSNRRLQQTQAQVEEVVDIIRVNVDKVLERDQ
Gorilla                MSAPAQPPAEGTEGTAPGGGPPGPPPNMTSNRRLQQTQAQVEEVVDIMRVNVDKVLERDQ
Orangutan              MSAPAQPPAEGTEGTAPGGGPPGPPPNLTSNRRLQQTQAQVEEVVDIMRVNVDKVLERDQ
PileatedGibbon         MSAPAQPPAEGTEGTAPGGGPPGPPPNMTSNRRLQQTQAQVEEVVDIMRVNVDKVLERDQ
WhiteHandedGibbon      MSAPAQPPAEGTEGTAPGGGPPGPPPNMTSNRRLQQTQAQVEEVVDIMRVNVDKVLERDQ
WhiteCheekedGibbon     MSAPAQPPAEGTEGTAPGGGPPGPPPNMTSNRRLQQTQAQVEEVVDIMRVNVDKVLERDQ
LeafMonkey             MSAPAQPPAEGTEGTAPGGGPPGPPPNLTSNRRLQQTQAQVEEVVDIIRVNVDKVLERDQ
Colobus                MSAPAQPPAEGTEGTAPGGGPPGPPPNLTSNRRLQQTQAQVEEVVDIIRVNVDKVLERDQ
BlackMangabey          MSAPAQPPAEGTEGTAPGGGPPGPPPNLTSNRRLQQTQAQVEEVVDIIRVNVDKVLERDQ
RhesusMacaque          MSAPAQPPAEGTEGTAPGGGPPGPPPNLTSNRRLQQTQAQVEEVVDIIRVNVDKVLERDQ
Marmoset               MSAPAQPPAEGTEGTAPGGGPPGPPPNMTSNRRLQQTQAQVEEVVDIMRVNVDKVLERDQ
TitiMonkey             MSAPAQLPAEGTEGTAPGGGPPGPPPNMTSNRRLQQTQAQVEEVVDIMRVNVDKVLERDQ
HowlerMonkey           MSAPAQPPAEGTEGTAPGGGPPGPPPNMTSNRRLQQTQAQVEEVVDIMRVNVDKVLERDQ
Bushbaby               MSAPAQPPAEGTEGTAPGGGPPGPPPNTTSNRRLQQTQAQVEEVVDIIRVNVDKVLERDQ
MouseLemur             MSAPAQPPAEGTEGTAPGGGPPGPPPNTTSNRRLQQTQAQVEEVVDIMRVNVDKVLERDQ
                       ****** ******************** *******************:************
                                                                     106
Human                  KLSELDDRADALQAGASQFESSAAKLKRKYWWKNCKMMIMLGAICAIIVVVIV
Chimp                  KLSELDDRADALQAGASQFESSAAKLKRKYWWKNCKMMIMLGAICAIIVVVIV
Bonobo                 KLSELDDRADALQAGASQFESSAAKLKRKYWWKNCKMMIMLGAICAIIVVVIV
Gorilla                KLSELDDRADALQAGASQFESSAAKLKRKYWWKNCKMMIMLGAICAIIVVVIV
Orangutan              KLSELDDRADALQAGASQFESSAAKLKRKYWWKNCKMMIMLGAICAIIVVVIV
PileatedGibbon         KLSELDDRADALQAGASQFESSAAKLKRKYWWKNCKMMIMLGAICAIIVVVIV
WhiteHandedGibbon      KLSELDDRADALQAGASQFESSAAKLKRKYWWKNCKMMIMLGAICAIIVVVIV
WhiteCheekedGibbon     KLSELDDRADALQAGASQFESSAAKLKRKYWWKNCKMMIMLGAICAIIVVVIV
LeafMonkey             KLSELDDRADALQAGASQFESSAAKLKRKYWWKNCKMMIMLGAICAIIVVVIV
Colobus                KLSELDDRADALQAGASQFESSAAKLKRKYWWKNCKMMIMLGAICTIIVVVIV
BlackMangabey          KLSELDDRADALQAGASQFESSAAKLKRKYWWKNCKMMIMLGAICAIIVVVIV
RhesusMacaque          KLSELDDRADALQAGASQFESSAAKLKRKYWWKNCKMMIMLGAICAIIVVVIV
Marmoset               KLSELDDRADALQAGASQFESSAAKLKRKYWWKNCKMMIMLGAICAIIVVVIV
TitiMonkey             KLSELDDRADALQAGASQFESSAAKLKRKYWWKNCKMMIMLGAICAIIVVVIV
HowlerMonkey           KLSELDDRADALQAGASQFESSAAKLKRKYWWKNCKMMIMLGAICAIIVVVIV
Bushbaby               KLSELDDRADALQAGASQFESSAAKLKRKYWWKNCKMMIMLGAICAIIVVVIV
MouseLemur             KLSELDDRADALQAGASQFESSAAKLKRKYWWKNCKMMIMLGAICAIIVVVIV
                       *********************************************:*******
```

Supplementary Figure 4. Sequence alignment of VAMP1 in selected primate species.

Protein sequences of exons 1-4 of the VAMP1 gene are aligned for the seventeen listed primate species. Out of the 113 amino acid positions shown, only four positions have accumulated non-synonymous mutations. Positions 7 and 106 have a single non-synonymous mutation in only one primate species (highlighted in green), where sites 28 and 48 are highly variable between species, having mutated multiple times over primate evolution (highlighted in yellow).
