# Supplementary material for: Widespread Sequence Variations in VAMP1 across Vertebrates Suggest a Potential Selective Pressure from Botulinum Neurotoxins
Source: PLoS Pathog. 2014 Jul 10;10(7):e1004177. doi: 10.1371/journal.ppat.1004177 (PMC4092145; doi:10.1371/journal.ppat.1004177)
Supplement: Figure S5 — Sequence alignment of VAMP2 in selected primate species. There are no non-synonymous mutations in VAMP2 across nine major primate species examined here, indicating that VAMP2 is highly conserved. (PDF) [file ppat.1004177.s005.pdf]

```
Human          MSATAATAPPAAPAGEGGPPAPPPNLTSNRRLQQTQAQVDEVVDIMRVNVDKVLERDQKL
Chimp          MSATAATAPPAAPAGEGGPPAPPPNLTSNRRLQQTQAQVDEVVDIMRVNVDKVLERDQKL
Gorilla        MSATAATAPPAAPAGEGGPPAPPPNLTSNRRLQQTQAQVDEVVDIMRVNVDKVLERDQKL
Orangutan      MSATAATAPPAAPAGEGGPPAPPPNLTSNRRLQQTQAQVDEVVDIMRVNVDKVLERDQKL
Gibbon         MSATAATAPPAAPAGEGGPPAPPPNLTSNRRLQQTQAQVDEVVDIMRVNVDKVLERDQKL
Rhesus         MSATAATAPPAAPAGEGGPPAPPPNLTSNRRLQQTQAQVDEVVDIMRVNVDKVLERDQKL
Marmoset       MSATAATAPPAAPAGEGGPPAPPPNLTSNRRLQQTQAQVDEVVDIMRVNVDKVLERDQKL
Bushbaby       MSATAATAPPAAPAGEGGPPAPPPNLTSNRRLQQTQAQVDEVVDIMRVNVDKVLERDQKL
MouseLemur     MSATAATAPPAAPAGEGGPPAPPPNLTSNRRLQQTQAQVDEVVDIMRVNVDKVLERDQKL
               ************************************************************

Human          SELDDRADALQAGASQFETSAAKLKRKYWWKNLKMMIILGVICAIILIIIIVYFST
Chimp          SELDDRADALQAGASQFETSAAKLKRKYWWKNLKMMIILGVICAIILIIIIVYFST
Gorilla        SELDDRADALQAGASQFETSAAKLKRKYWWKNLKMMIILGVICAIILIIIIVYFST
Orangutan      SELDDRADALQAGASQFETSAAKLKRKYWWKNLKMMIILGVICAIILIIIIVYFST
Gibbon         SELDDRADALQAGASQFETSAAKLKRKYWWKNLKMMIILGVICAIILIIIIVYFST
Rhesus         SELDDRADALQAGASQFETSAAKLKRKYWWKNLKMMIILGVICAIILIIIIVYFST
Marmoset       SELDDRADALQAGASQFETSAAKLKRKYWWKNLKMMIILGVICAIILIIIIVYFST
Bushbaby       SELDDRADALQAGASQFETSAAKLKRKYWWKNLKMMIILGVICAIILIIIIVYFST
MouseLemur     SELDDRADALQAGASQFETSAAKLKRKYWWKNLKMMIILGVICAIILIIIIVYFST
               ********************************************************
```

Supplementary Figure 5. Sequence alignment of VAMP2 in selected primate species.

There are no non-synonymous mutations in VAMP2 across nine major primate species examined here, indicating that VAMP2 is highly conserved.
