# Supplementary material for: Widespread Sequence Variations in VAMP1 across Vertebrates Suggest a Potential Selective Pressure from Botulinum Neurotoxins
Source: PLoS Pathog. 2014 Jul 10;10(7):e1004177. doi: 10.1371/journal.ppat.1004177 (PMC4092145; doi:10.1371/journal.ppat.1004177)
Supplement: Figure S6 — Geographic distribution of major primate species. The geographic distribution of all seventeen primate species examined in Fig. 4C was color-coded and plotted on the map. The primates with M48 in VAMP1 are marked in blue, whereas the primates with I48 in VAMP1 are marked in red. (PDF) [file ppat.1004177.s006.pdf]

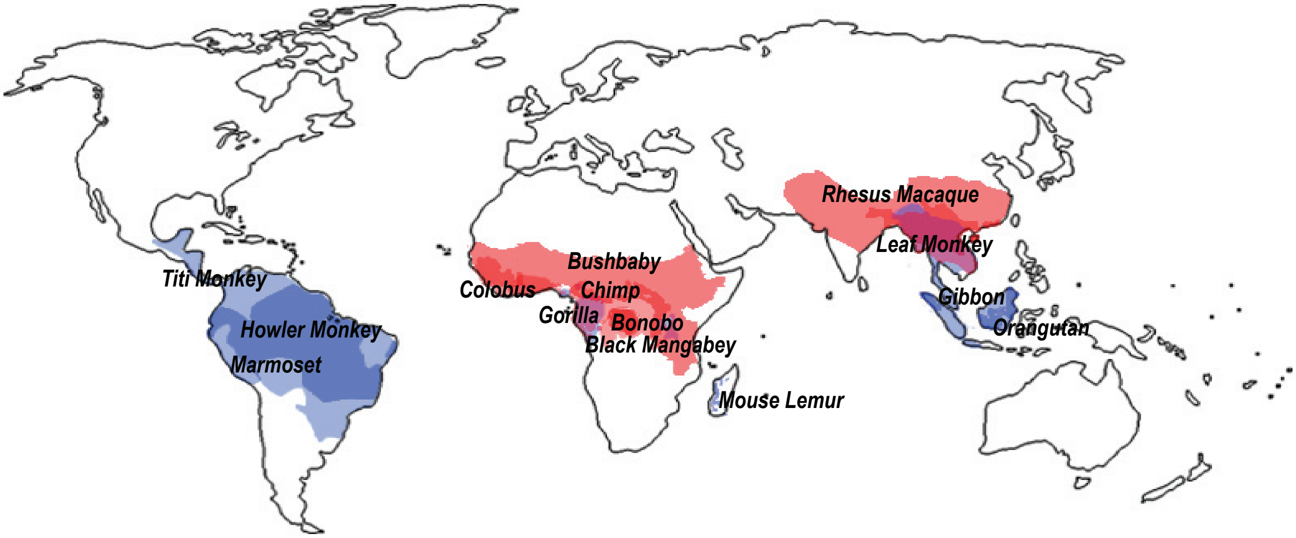

Supplementary Figure 6. Geographic distribution of major primate species.

The geographic distribution of all seventeen primate species examined in Fig. 4C was color-coded and plotted on the map. The primates with M48 in VAMP1 are marked in blue, whereas the primates with I48 in VAMP1 are marked in red.
